# Annexin A5 is the Most Abundant Membrane-Associated Protein in Stereocilia but is Dispensable for Hair-Bundle Development and Function

**DOI:** 10.1038/srep27221

**Published:** 2016-06-02

**Authors:** Jocelyn F. Krey, Meghan Drummond, Sarah Foster, Edward Porsov, Sarath Vijayakumar, Dongseok Choi, Karen Friderici, Sherri M. Jones, Alfred L. Nuttall, Peter G. Barr-Gillespie

**Affiliations:** 1 Oregon Hearing Research Center, Oregon Health & Science University, Portland, OR 97239, USA; 2 Vollum Institute, Oregon Health & Science University, Portland, OR 97239, USA; 3 Department of Microbiology & Molecular Genetics, Department of Pediatrics & Human Development, Michigan State University, East Lansing, MI 48824, USA; 4 Department of Special Education & Communication Disorders, University of Nebraska-Lincoln, Lincoln, NE 68583, USA; 5 School of Public Health, Oregon Health & Science University, Portland, OR 97239, USA

## Abstract

The phospholipid- and Ca^2+^-binding protein annexin A5 (ANXA5) is the most abundant membrane-associated protein of ~P23 mouse vestibular hair bundles, the inner ear’s sensory organelle. Using quantitative mass spectrometry, we estimated that ANXA5 accounts for ~15,000 copies per stereocilium, or ~2% of the total protein there. Although seven other annexin genes are expressed in mouse utricles, mass spectrometry showed that none were present at levels near ANXA5 in bundles and none were upregulated in stereocilia of *Anxa5*^−/−^ mice. Annexins have been proposed to mediate Ca^2+^-dependent repair of membrane lesions, which could be part of the repair mechanism in hair cells after noise damage. Nevertheless, mature *Anxa5*^−/−^ mice not only have normal hearing and balance function, but following noise exposure, they are identical to wild-type mice in their temporary or permanent changes in hearing sensitivity. We suggest that despite the unusually high levels of ANXA5 in bundles, it does not play a role in the bundle’s key function, mechanotransduction, at least until after two months of age in the cochlea and six months of age in the vestibular system. These results reinforce the lack of correlation between abundance of a protein in a specific compartment or cellular structure and its functional significance.

Understanding how the sensory cells of the inner ear, hair cells, act to encode auditory or vestibular stimuli^1^,^2^ requires deep understanding of the biochemical and physiological functions of the hundreds of proteins that make up the sensory hair bundle^3^. Each bundle consists of ~100 actin-rich stereocilia, which project from the apical surface of the cell body; deflection of the bundle by mechanical stimuli opens cation-selective transduction channels that admit K^+^ and Ca^2+^ and depolarize the hair cell. Many of the proteins that are highly enriched in bundles are encoded by deafness genes, reinforcing their functional significance^3^. Less is known about other proteins that are enriched or abundant in bundles. For example, bundles from the chick utricle, a vestibular organ that detects linear acceleration, have ~11,000 molecules of ANXA5 (annexin A5) per stereocilium and almost no other annexins^3^. While this level makes ANXA5 the most abundant membrane-associated protein in chick bundles, its functional significance there is not known.

Annexins are soluble proteins that, in the presence of elevated Ca^2+^, are recruited to membranes containing negatively charged phospholipids. Vertebrates express 12 annexin genes (A1–A11 and A13), which have both overlapping and exclusive biological activities. Famous in its role as an exogenous probe for extracellularly exposed phosphatidylserine, a marker of apoptosis, ANXA5 is one of the best-characterized annexins. Atomic force microscopy shows that after binding to membranes, ANXA5 assembles into a two-dimensional crystalline array^4^, which is capable of trapping and immobilizing membrane proteins^5^. Many roles have been proposed for annexins, including protein scaffolding, membrane aggregation, exocytosis and endocytosis regulation, control of apoptosis, coagulation regulation, and membrane remodeling^6^,^7^. The evidence for annexin participation in these processes is often less than compelling, however. By contrast, there is strong evidence for the participation of ANXA5 in resealing plasma-membrane disruptions^8^,^9^. The local influx of unphysiologically high Ca^2+^ levels that occurs after membrane rupture is sufficient to promote ANXA5 binding to torn membrane edges; two-dimensional arrays of ANXA5 are formed, which prevents expansion of the membrane wound and promotes resealing^8^,^9^. Other annexins may share this rupture-resealing activity^10^,^11^.

*Anxa5*^−/−^ mice are phenotypically normal with most physiological assays^12^. Litter and fetal size of *Anxa5*^−/−^ mice are both reduced, however, but only if the mother was *Anxa5*^−/−^; maternal ANXA5 is apparently crucial for maintaining intact placental circulation^13^. Because there are 11 other annexin proteins expressed in mouse tissues, however, the lack of more extensive phenotypes could arise because other annexins (or other protein families) substitute for ANXA5 in most functions.

We found that hair bundles of mouse utricles are also rich in ANXA5, which was the most abundant membrane-associated protein there and whose concentration was considerably greater than that of other annexins in bundles. Because of the lack of other annexin isoforms that might compensate for the loss of ANXA5, bundles might be a particularly good place to discern the function of this protein. Mature *Anxa5*^−/−^ mice had normal vestibular and auditory function, however, and were not sensitive to noise damage. Mass spectrometry confirmed that no other annexins compensated for the loss of ANXA5 in hair bundles. Any role for ANXA5 is likely to be in the hair-cell soma, not bundle, and the other annexins (ANXA2 and ANXA6) highly expressed in the soma may be capable of taking the place of ANXA5. ANXA5 may be present in stereocilia because of favorable environmental conditions, not because there is a need for annexins for function of this structure.

## Results

### ANXA5 is abundant in hair bundles

To determine the concentration of ANXA5 in mouse hair bundles, we isolated bundles from utricles of postnatal days 4 to 6 (“P5”; developing) and P21-P25 (“P23”; young adult) mice using the twist-off technique^14^,^15^,^16^; we then characterized proteins of bundles with shotgun mass spectrometry and label-free quantitation^17^. Measurement with MaxQuant^18^ and the iBAQ algorithm^19^ of areas of peptide extracted ion chromatograms, which were collected with an Orbitrap mass spectrometer, allowed us to comprehensively profile protein abundance in the bundle^17^,^20^. We calculated each protein’s relative molar abundance in isolated bundles and whole utricle^20^.

**Figure 1:**
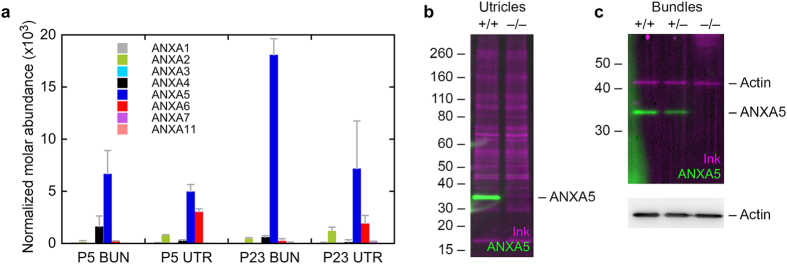
Protein chemistry analysis of annexins in vestibular hair cells. (**a**) Annexin paralog expression in developing and mature mouse utricle and hair bundles using quantitative mass spectrometry. The y-axis indicates the relative fraction (concentration) of each annexin in the appropriate sample. Samples were P5 hair bundles (P5 BUN), P5 whole utricle (P5 UTR), P23 hair bundles (P23 BUN), and P23 whole utricle (P23 UTR). Four replicates each of 100 ear-equivalents (bundles) or 10 utricles were analyzed. Mean ± SD are plotted. (**b**) Protein immunoblotting of ANXA5 in P23 wild-type and *Anxa5*-mutant utricles. One utricle was analyzed for each genotype; the immunoblot was first stained with India ink (magenta) to visualize total protein, then was probed with anti-ANXA5 (green). The band of the correct molecular mass (~33 kD) seen in wild-type utricles was completely absent from the knockout utricles. (**c**) Protein immunoblotting of ANXA5 in P23 wild-type and *Anxa5*-mutant hair bundles (7 ear-equivalents per age). Genotypes indicated above lanes. Because of its abundance in bundles, actin was the only protein readily detected by the ink stain; ANXA5 was detected by immunoblotting in wild-type and heterozygous bundles, but not in homozygous knockout bundles. Actin was detected by immunoblotting at similar levels in all three lanes.

Like in chicken hair bundles, ANXA5 was the most abundant membrane-associated protein of P5 mouse utricle hair bundles (Fig. 1a); assuming that there are 400,000 copies of actin per stereocilium, there are ~3,500 ANXA5 molecules per stereocilium. This concentration is greater than that of the other annexin isoforms in bundles, which collectively account for only 1000 molecules per stereocilium. ANXA5 increases considerably in abundance as mouse bundle development proceeds; by P23 there are ~15,000 ANXA5 molecules per 400,000 actins, and still only 1000 other annexins (Fig. 1).

Nevertheless, hair bundles account for <1% of the volume of the mouse utricle^20^. By multiplying the contribution of bundles or somas to the utricle by the relative fractions of ANXA5 in those fractions, we estimated that >95% of the ANXA5 in utricles is in the combined somas of hair cells and supporting cells, not in bundles, even at P23. Moreover, while ANXA5 was also the most abundant annexin in whole utricle, ANXA2 and ANXA6 were present at substantial levels (Fig. 1a). Altogether, the non-ANXA5 annexins accounted for 45% of the total annexin abundance in P5 utricles and 34% in P23 utricles. Thus while ANXA5 is by far the most abundant annexin in bundles, other annexins are present in significant amounts in utricles.

### Localization of ANXA5 in mouse auditory and vestibular hair cells

**Figure 2:**
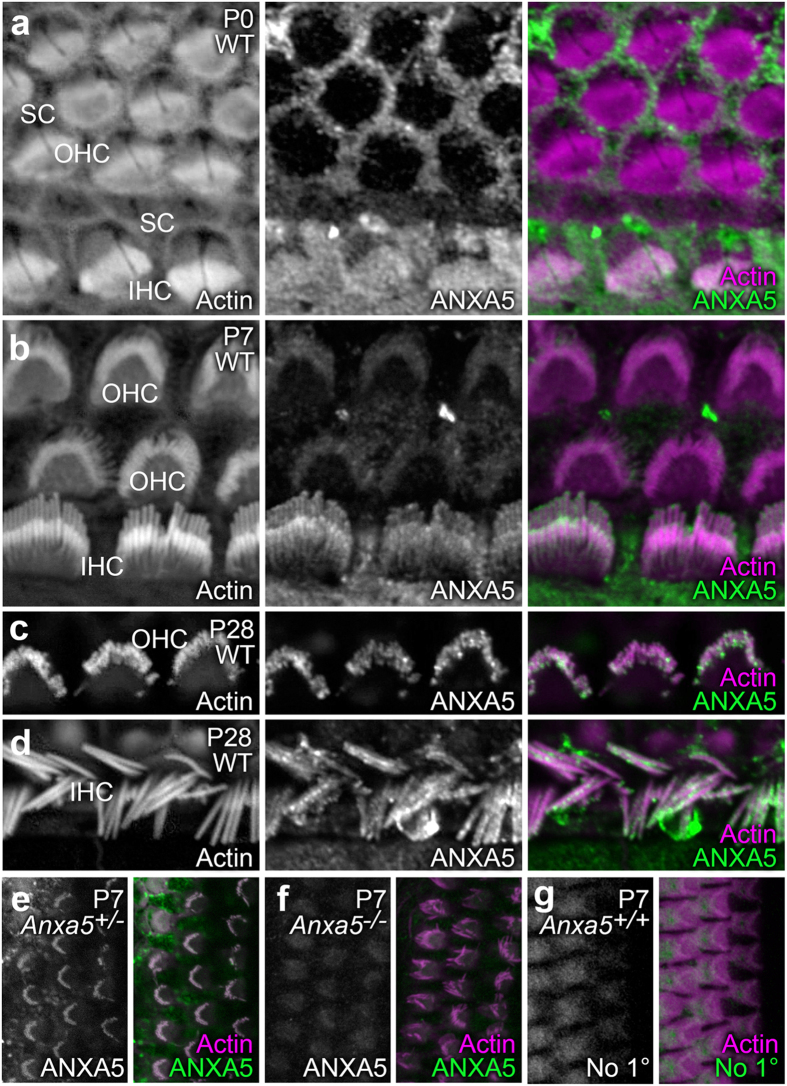
Localization of ANXA5 in organ of Corti stereocilia shifts during postnatal development. For (**a–e**) samples were labeled with rhodamine-phalloidin (left panels and magenta in right-panel merges) and anti-ANXA5 (middle panels and green in right-panel merges). (**a**) At P0, inner hair cell (IHC) stereocilia had ANXA5 labeling throughout the bundle, whereas labeling was absent in outer hair cells (OHC). Supporting cell (SC) apical surfaces were strongly labeled. (**b**) At P7, ANXA5 was visible in OHC stereocilia and displays distinct localization to the tips, as well as periphery of IHC stereocilia. (**c**) At P28, ANXA5 as observed at stereocilia tips and shafts of OHCs. (**d**) ANXA5 stereocilia tips and shafts of IHCs at P28. (**e–g**) Labeling controls; antibody channel alone is in the left panel, and the merge with the phalloidin channel is in the right panel. (**e**) *Anxa5*^*+/−*^ heterozygote cochlea, labeled with anti-ANXA5. (**f**) *Anxa5*^−/−^ homozygote cochlea, labeled with anti-ANXA5. (**g**) Wild-type cochlea, labeled with secondary antibody only. Background labeling only was seen in Anxa5-null and secondary-only controls. Panel full widths: a–e, 20 μm; f,g, 30 μm.

We used *Anxa5*^−/−^ mice^12^ to determine the roles of ANXA5 in the inner ear, first using these mice to confirm the specificity of the antibody used for immunolocalization. This antibody detected a protein of the correct molecular mass in P23 wild-type utricles, which was absent in knockout animals (Fig. 1b). ANXA5 was also readily detected in P23 hair bundles, where its expression level was proportional to the number of wild-type alleles (Fig. 1c). Finally, the antibody labeled hair cells of P0 wild-type but not *Anxa5*^−/−^ mice (Fig. 2e–f).

In the P0 mouse cochlea, ANXA5 was abundant in apical microvilli of supporting cells, with some signal in inner hair cells (IHC; Fig. 2a). By P7, however, the supporting-cell signal declined and the signal in IHCs was more prominent, with a concentration at IHC stereocilia tips (Fig. 2b). A weak signal was seen in outer hair cells (OHC). By P28, the stereocilia signal in both OHCs (Fig. 2c) and IHCs (Fig. 2d) was much greater; the signal was punctate, but localized towards stereocilia tips.

ANXA5 binding to surface-exposed lipids is used as a marker for apoptosis^21^. Like many other cell types, in response to stressful conditions, hair cells translocate phosphatidylserine; these damaged hair cells can be strongly labeled by exogenous ANXA5 (ref. 22). Nevertheless, ANXA5 was not present on the extracellular leaflet of hair cells (Supplementary Fig. S1). In addition, while a previous report demonstrated an isoform-specific interaction between ANXA5 and ACTG1 (ref. 7), a component of the stereocilia cytoskeleton, we saw no change in ACTG1 immunolabeling in *Anxa5*^−/−^ mice (Supplementary Fig. S2).

**Figure 3:**
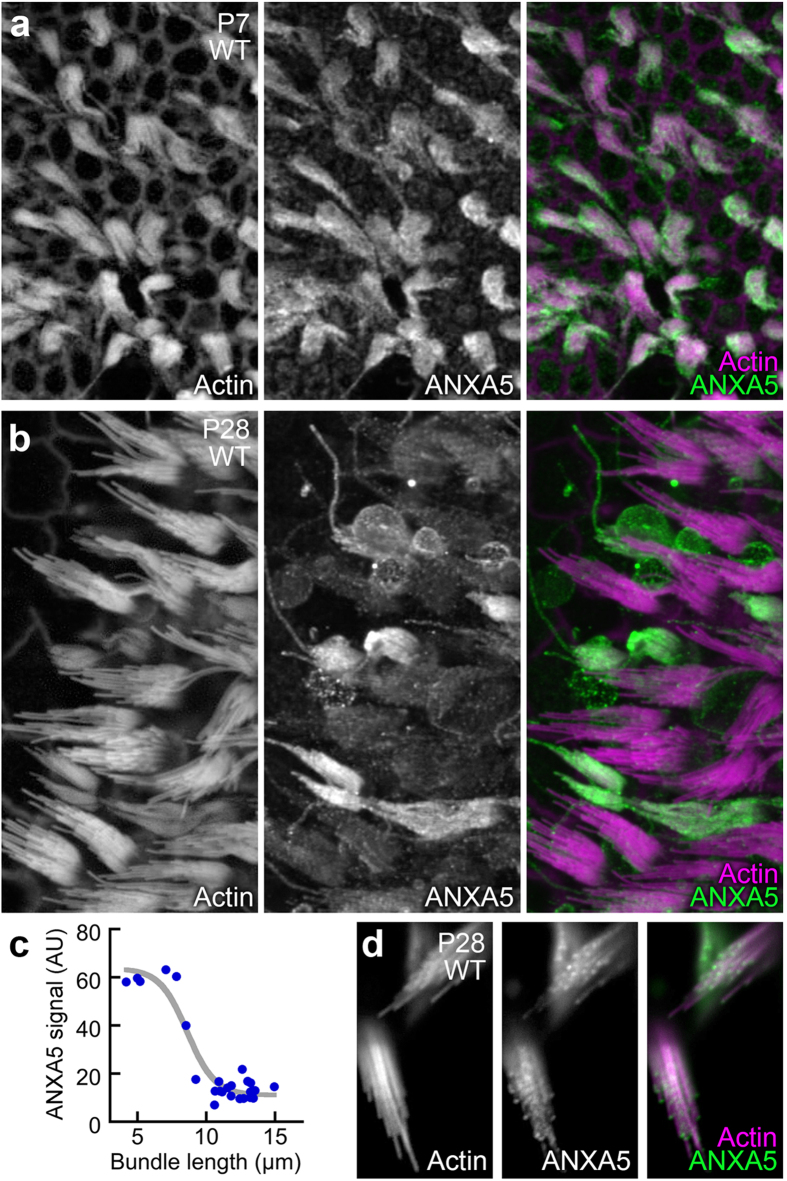
Distribution of ANXA5 in vestibular stereocilia changes with age. Samples were labeled with rhodamine-phalloidin (left panels and magenta in right-panel merges) and anti-ANXA5 (middle panels and green in right-panel merges). (**a**) In P7 saccule, hair bundles of all hair cells are labeled uniformly. (**b**) In P28 saccule, differences between neighboring bundles are apparent, with shorter bundles having higher ANXA5 immunoreactivity. (**c**) Relationship between the length of the tallest stereocilium in a saccular bundle and the ANXA5 fluorescence signal (AU, arbitrary units). Data were fit with a logistic function: y = 64−53/(1 + e^−(x−8.6)^). (**d**) High-magnification views of several hair cells with short bundles. Labeling is strong at stereocilia tips. Panel full widths: a, 180 μm; b, 130 μm; d, 20 μm.

In the mouse saccule, another vestibular organ that detects linear acceleration, ANXA5 labeling was uniform in P7 hair bundles (Fig. 3a). By P28, however, the ANXA5 signal varied between bundles; shorter bundles were ensheathed in ANXA5, while taller bundles had much less ANXA5 (Fig. 3b). Bundles with high levels of ANXA5 were all 4–7 μm tall, while ones with low levels were >9 μm tall (Fig. 3c); ANXA5 in these taller bundles was clearly concentrated at stereocilia tips (Fig. 3d).

### No upregulation of annexins in *Anxa5* ^−/−^ mice

We used shotgun mass spectrometry with an ion-trap mass spectrometer to determine whether the hair-bundle levels of annexin isoforms or any other protein were elevated in *Anxa5*^−/−^ mouse utricles. Bundles were isolated from *Anxa5*^−/−^ mice with an efficiency similar to that of wild-type bundles. We then used shotgun mass spectrometry with label-free detection to quantify proteins of bundles, in this case using relative molar intensities derived from summing MS2 intensities for all peptides detected for each protein^17^. Because we used a less-sensitive instrument than that used for the experiment described in Fig. 1, used three rather than four replicates, and used bundles from only ~30 rather than ~100 ears per replicate, we detected fewer proteins overall.

**Figure 4:**
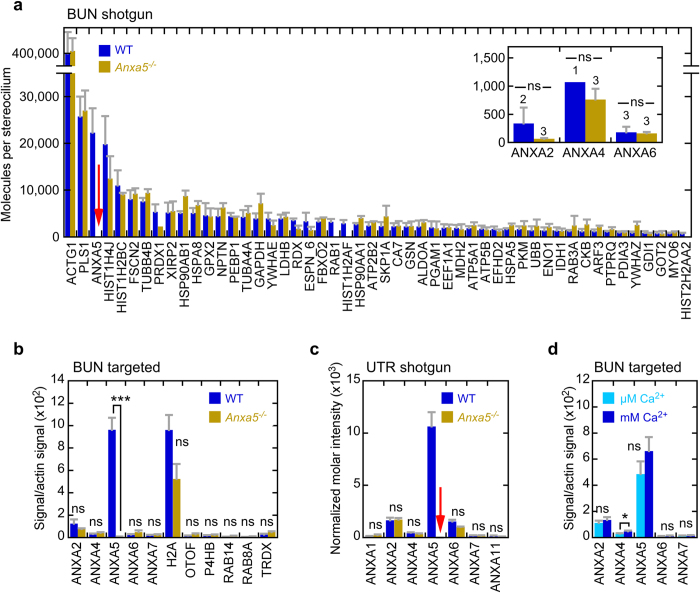
*Anxa5*^−/−^ inner-ear proteomics in P23 mice. (**a**) Comparison of wild-type and *Anxa5*^−/−^ hair-bundle proteomes, determined with shotgun proteomics and quantified with MS2 intensities. Three biological replicates of 32 ear-equivalents of bundles were used for each condition. Relative molar intensity was calculated for each protein, and was converted to molecules per stereocilium assuming 400,000 actin molecules per stereocilium in wild-type bundles. The mean ± SD for the 50 most abundant proteins in wild-type bundles are displayed. Note the absence of signal for ANXA5 in *Anxa5*^−/−^ mice (red arrow). Inset, molecules per stereocilium for other annexins; no significant changes. (**b**) Targeted proteomics analysis of hair bundles of wild-type and *Anxa5*^−/−^ mice. All annexins and key differentially-expressed proteins from the shotgun experiment were quantified. For each condition, four biological replicates of 10 ear-equivalents each were used. Mean ± SEM; ***p < 0.001; ns, not significant. (**c**) Shotgun proteomics with MS2 quantitation of wild-type and *Anxa5*^−/−^ whole utricles. ANXA5 was not detected in *Anxa5*^−/−^ mice (red arrow); none of the other annexins changed significantly in abundance. For each condition, two biological replicates with two technical replicates each of 0.5 utricles each were used. Mean ± SEM. (**d**) Targeted proteomics indicates that bundles isolated in the presence of high levels of Ca^2+^ have a minimal, non-significant increase in ANXA5. For each condition, four biological replicates of 5 ear-equivalents each were used. Mean ± SEM; *p < 0.05.

With the exception of ANXA5, the hair-bundle proteomes of wild-type and *Anxa5*^−/−^ mice were very similar (Fig. 4a; Supplementary Dataset 1). Of the 218 proteins consistently detected in both wild-type and *Anxa5*^−/−^ bundles, none changed significantly in expression level after adjusting for false-discovery rates due to multiple tests^23^ (Supplementary Dataset 1; all adjusted p-values were >0.7). Two histone groups (HIST1H2AF and H2AFJ; HIST2H2AA2, H2AFX, and HIST2H2AB) were detected only in wild-type mice, as was one RAB group (RAB1, RAB1B, RAB8A, RAB8B, RAB10, RAB13, and RAB35) and RAB14. Only one protein was only detected in *Anxa5*^−/−^ mice (CAMK2D).

Because statistical tests for significance of up- or down-regulation are not applicable when proteins are only detected in one set of samples, we instead measured the concentration of several of these proteins, as well as others from an earlier analysis of the same data, using targeted proteomics^24^. We assayed 2–5 peptides from each protein using parallel reaction monitoring (PRM), a targeted method that is more sensitive and quantitative than shotgun proteomics^25^. None of the monitored proteins were significantly up- or down-regulated in *Anxa5*^−/−^ mice (Fig. 4b). Finally, none of the annexins detected in utricular bundles changed in abundance in *Anxa5*^−/−^ mice as determined either by shotgun proteomics (Fig. 4a, inset) or by targeted proteomics (Fig. 4b). Together the data suggest that *Anxa5*^−/−^ bundles were very similar to wild-type bundles, with the exception that ANXA5 was missing.

We also measured the whole-utricle proteomes of wild-type and *Anxa5*^−/−^ mice (Supplementary Dataset 1). Of the 820 proteins detected in at least three of four wild-type and three of four *Anxa5*^−/−^ samples, after correction for multiple tests, none were significantly up- or down-regulated in *Anxa5*^−/−^ utricles (Supplementary Dataset 1; all p-values, adjusted false-discovery rate, were >0.3). Similarly, with the exception of ANXA5, none of annexins changed in abundance in the mutant utricles (Fig. 4c). Together our proteomics results suggest that there was no compensation for the loss of ANXA5 in the knockout animals.

### No effect of Ca^2+^ on ANXA5 targeting to hair bundles

A plausible hypothesis for the presence of ANXA5 in stereocilia is that the protein is recruited there when high levels of Ca^2+^ enter through open transduction channels, especially when the bathing medium contains high levels of Ca^2+^ (e.g., our normal dissection solution). ANXA5 requires millimolar levels of Ca^2+^ for maximal binding to membranes^26^. Stereocilia are normally exposed to a solution called endolymph, which has an unusual ionic composition; normal endolymph Ca^2+^ is ~25 μM in cochlea^27^ and only an order of magnitude higher in vestibular organs^28^, not millimolar. Although Ca^2+^ can be at higher levels at intracellular sites of entry because of the highly negative membrane potential, diffusion should reduce its levels well below that needed for ANXA5 binding throughout the stereocilia. When external Ca^2+^ is millimolar, however, such as during tissue isolation in the presence of 1.26 mM Ca^2+^, the stereocilia concentration might be elevated throughout. To test this hypothesis, we isolated hair bundles using our normal buffer solution containing no added Ca^2+^, which has 3.1 ± 0.1 μM Ca^2+^ derived from water, and compared the level of annexins there to levels present in bundles isolated using our normal millimolar Ca^2+^ levels (measured at 1.27 ± 0.13 mM). Using PRM to accurately measure protein abundance, we found a small (<40%) but statistically significant increase in the amount of ANXA4 that was present in bundles that were isolated with millimolar Ca^2+^ as compared with those isolated with no added Ca^2+^ (Fig. 4d). ANXA4 was present at a very low level, however. The other annexins, including ANXA5, did not change significantly in abundance (p > 0.2 for each).

### Vestibular and auditory function is normal in *Anxa5* ^−/−^ mice

**Figure 5:**
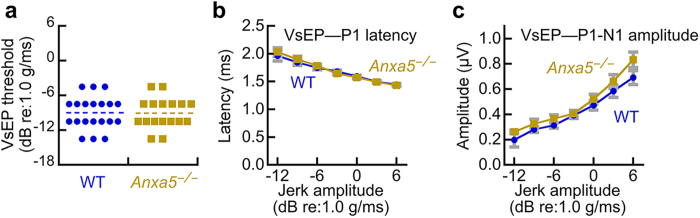
Vestibular function of wild-type and *Anxa5*^−/−^ mice. (**a**) Vestibular evoked potential thresholds. There was no difference in the mean values (dashed lines) between wild-type and *Anxa5*^−/−^ mice. (**b**) P1 latency values; no differences between wild-type and *Anxa5*^−/−^ mice. (**c**) P1-N1 amplitudes; no differences between wild-type and *Anxa5*^−/−^ mice. WT mice averaged 5.7 months old (range 3.9 to 7.1 months), and *Anxa5*^−/−^ averaged 5.4 months old (range 4.7 to 6.1 months). All mice were on a C57BL/6 background.

Measurement of vestibular evoked potentials (VsEPs) allows for sensitive, non-invasive measurement of the function of vestibular end-organs and their neural pathways^29^. Several parameters can be measured; latencies provide a measure of the timing of primary afferent neural activation, amplitude reflects the number of cells responding to the stimulus and the degree of synchronization among discharging neurons, and thresholds measure the general sensitivity of the utricle and saccule, the vestibular end organs that detect linear acceleration (Fig. 5a). Despite the high levels of ANXA5 in wild-type stereocilia (Fig. 1), vestibular function of *Anxa5*^−/−^ mice at ~6 months of age as assessed with VsEPs was normal (Fig. 5). No difference between wild-type and *Anxa5*^−/−^ mice was observed for any VsEP parameter, including VsEP thresholds, the amplitude-intensity function, or the P1 latency-intensity function (Fig. 5). No gross behavioral defects were seen with *Anxa5*^−/−^ mice, e.g., the mice did not show circling behavior and could swim. Vestibular function was thus normal in *Anxa5*^−/−^ mice.

**Figure 6:**
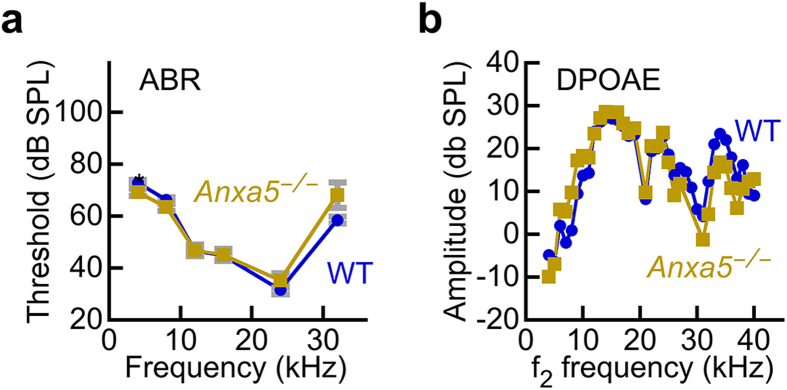
Auditory function of wild-type and *Anxa5*^−/−^ mice. (**a**) Auditory brainstem response. No difference in ABR thresholds between wild-type and *Anxa5*^−/−^ mice. (**b**) Distortion-product otoacoustic emissions. No difference in DPOAE amplitudes between wild-type and *Anxa5*^−/−^ mice. WT mice averaged 2.3 months old (range 2.3 to 2.4 months), and *Anxa5*^−/−^ averaged 2.3 months old (range 2.3 to 2.5 months). All mice were on a C57BL/6 background.

Similarly, auditory function was also normal in *Anxa5*^−/−^ mice that were ~2 months old. While the C57BL/6 strain we used as background does exhibit age-related hearing loss, loss of hearing only occurs after ~12 months of age^30^. Auditory brainstem response (ABR) measurements detect evoked potentials in the auditory nerve and central auditory pathways in response to auditory stimuli. Overall sensitivity of the inner ear is often assessed by measuring the threshold where electrical responses corresponding to wave I of the ABR response can just be detected. ABR wave I thresholds were not significantly different between wild-type and *Anxa5*^−/−^ mice (Fig. 6), except at 4 kHz where the *Anxa5*^−/−^ mice had slightly better sensitivity (p = 0.03). Likewise, levels of distortion-product otoacoustic emissions (DPOAE), which measures the function of OHC, were nearly identical for wild-type and *Anxa5*^−/−^ mice (Fig. 6). Together these datasets show that auditory function is normal in *Anxa5*^−/−^ mice.

### Temporary and permanent threshold shift following noise damage is unaltered in *Anxa5* ^−/−^ mice

**Figure 7:**
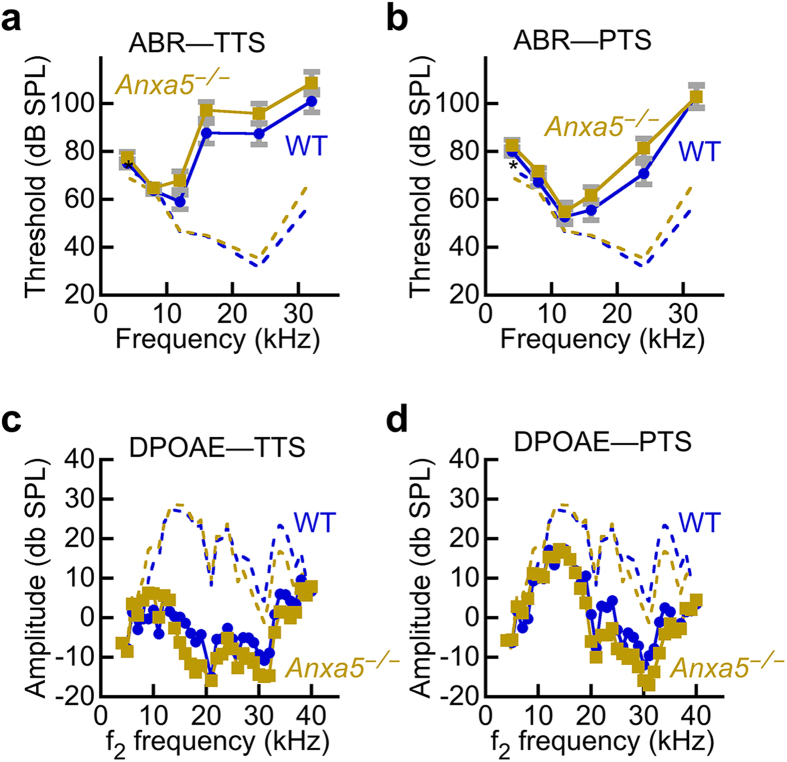
ANXA5 does not protect against noise damage. Mice were tested for baseline auditory function (dashed lines, derived from the data of Fig. 6), then were subjected to noise damage that elicited a robust temporary threshold shift (TTS); the noise damage was sufficiently large that auditory function did not return to baseline, thus producing a permanent threshold shift (PTS). (**a**) Auditory brainstem response during temporary threshold phase. (**b**) ABR during permanent threshold phase. (**c**) Distortion-product otoacoustic emission amplitudes during TTS phase. (**d**) DPOAE during PTS phase. There was no difference between wild-type and *Anxa5*^−/−^ mice in any of the four conditions. The animals were the same individuals at the same ages as in Fig. 6.

ANXA5 could plausibly protect against damage to hair cells, and damage from noise is common and well characterized. To assess whether ANXA5 protects against damage from noise exposure, mice were exposed to a loud-sound protocol using a 96 dB SPL stimulus at 8–16 kHz for 2 hours; this treatment results in a moderate temporary loss of auditory function, followed by a small-to-moderate amount of permanent cochlea damage and loss of hearing sensitivity. As assessed by ABR and DPOAE measurements, *Anxa5*^−/−^ and wild type mice were indistinguishable in their temporary and permanent hearing loss after noise damage (Fig. 7). These results suggest that ANXA5 does not assist in recovery of hair cells after exposure to noise.

## Discussion

Despite its high levels in stereocilia, ANXA5 does not appear to play a unique functional role for the auditory and vestibular systems. Although we are conditioned to believe that a molecule that is abundant in a cell or tissue must be involved in a critical process that necessitates its high level of expression, our data show that this conclusion is not necessarily correct. Certainly, the converse is not true—demonstrably, proteins present at low levels in cells can be highly functionally significant. While ANXA5 may participate in some way in hair-cell function, we saw no defects using standard vestibular and auditory tests; as these tests assess the fundamental roles of the inner ear, these negative results suggest that any function of ANXA5 in hair cells must be restricted to highly specialized conditions.

While quantitation using MS1 or MS2 intensities is consistent from protein to protein only on average, our quantitative results were strengthened by the similarity of estimated ANXA5 abundance in mouse and chick utricle hair bundles. At the protein level, mouse and chick ANXA5 are only 77% identical; moreover, out of ~40 tryptic peptides present in each, only seven are shared between them. The similarity in protein abundance in mouse and chick bundles, quantified by very different sets of peptides, suggests that the abundance estimates are reasonably accurate. Moreover, ANXA5 is relatively large (36 kD) and accounts for >1% of the total bundle protein, both features that bias the molecule towards accurate detection using mass spectrometry^17^. These considerations indicate that the high concentration we measure for mouse ANXA5 in stereocilia is accurate.

The proteomics experiments demonstrated that ANXA5 is the most abundant membrane-associated protein in isolated hair bundles; indeed, it is more abundant than other membrane-associated proteins, including all other annexins, in the whole utricle as well. Still, while stereocilia have substantial amounts of ANXA5, its single-molecule cross-sectional area is ~30 nm (ref. 4) and ANXA5 should cover only ~10% of the inner leaflet surface area. This degree of coverage is not surprising, however, as ANXA5 binds to anionic phospholipids^26^,^31^, and summed anionic phospholipids account for ~9% of total bundle lipids^32^.

The proteome of *Anxa5*^−/−^ mouse hair bundles shows very few changes in comparison with that of wild-type mice. In shotgun proteomics, we detected ANXA2, ANXA4, and ANXA6, but they were all far less abundant than ANXA5 and none of them changed significantly in *Anxa5*^−/−^ bundles. Moreover, no new annexin paralog was recruited to bundles in response to the loss of ANXA5, suggesting that no annexin is likely to take over any bundle roles of ANXA5. In addition, there is no evidence that any other class of proteins is recruited to stereocilia to serve a functional role previously filled by ANXA5. Similarly, except for ANXA5, there was no change in the abundance of any of the annexins—or any other protein—in the whole utricle, which includes both hair cells and supporting cells. Thus our results show that when ANXA5 is lost, hair cells have no change in protein expression to compensate for this protein’s absence.

Despite the lack of compensation, the auditory and vestibular systems both appear to operate normally in the absence of ANXA5. Vestibular evoked potential measurements are a more sensitive and objective measure of gravity receptor function than behavioral tests, and the lack of any significant difference between wild-type and *Anxa5*^−/−^ mice suggests that ANXA5 is not involved in mechanotransduction or synaptic transmission in vestibular hair cells. Likewise, auditory brainstem response measurements assay the sensitivity of the auditory system overall, and the lack of differences between genotypes indicates that ANXA5 is not involved in auditory transduction and transmission. That conclusion is reinforced by the lack of a difference between distortion-product otoacoustic emissions in wild-type and *Anxa5*^−/−^ mice; DPOAE amplitudes are a sensitive measure of mechanotransduction in outer hair cells, and the results reported here indicate that ANXA5 plays no apparent role there.

A plausible role for ANXA5 is protection against membrane lesions in stereocilia that could result from environmental damage, such as noise. Our noise-damage experiments argue against that role for ANXA5, however, as both temporary and permanent threshold shifts following noise exposure were not significantly altered by the absence of ANXA5. Damage to the soma membrane could be ameliorated by ANXA5 recruitment, but such damage does not seem to be present in our noise-damage paradigm. Together the results from our examination of inner-ear function in *Anxa5*^−/−^ mice suggest either that any cellular function that ANXA5 carries out is subtle, i.e., not measured in our assays, or that other annexins (or other non-annexin proteins) can substitute for ANXA5.

Many genes involved in auditory function do not display an acute deafness phenotype when knocked out, only revealing their significance for the system through progressive or age-related hearing loss. Because *Anxa5* might fall into this category, future studies examining the hearing of *Anxa5*^−/−^ mice—on a better-hearing background than C57BL/6, such as CBA—could tease out an auditory functional role in hair-cell maintenance. Vestibular function is not affected in the C57BL/6 strain^33^, however, and no age-related vestibular loss was noted, at least up to six months of age. Moreover, our experiments were designed to test a substantial role for ANXA5, which was expected given its abundance in utricle hair bundles. Clearly ANXA5 does not play a fundamental role in constructing a normal hair bundle or in its operation.

Given the abundance of ANXA2 and ANXA6 in the whole utricle, why do they not concentrate in stereocilia too? The most likely explanation arises from the cooperativity of annexin binding to phospholipids^34^, so that ANXA5—present at higher levels and more easily undergoing clustering—may outcompete the other annexins in hair bundles. In addition, while ANXA2 and ANXA6 also bind to anionic phospholipids in the presence of Ca^2+^, binding affinities and lipid specificities vary^6^ and so ANXA5 may bind more avidly to stereocilia binding sites. The lack of recruitment of other annexins in *Anxa5*^−/−^ mouse bundles is consistent with this interpretation. Finally, external Ca^2+^ and open transduction channels apparently do not influence whether ANXA5 or the other annexins concentrate in hair bundles. Together the data indicate that ANXA5 concentrates in stereocilia because of favorable environmental conditions, likely due to specific bundle lipids, and that ANXA5 does not compete with other annexins for the stereocilia binding sites.

Our results reinforce the conclusion that abundance of a protein does not always imply function. ANXA5 is present at high levels of stereocilia, yet auditory and vestibular tests that require stereocilia mechanotransduction show that *Anxa5*^−/−^ and wild-type mice behave identically. ANXA5 may play a “hitchhiker” role^35^ and partition into stereocilia because of favorable conditions there. Although ANXA5 is abundant in stereocilia, only a small amount of the total annexin in hair cells is present there, so the function of this protein family may be exclusively in the soma. If so, the substantial presence of other annexins may be sufficient to compensate for any loss of ANXA5 in the soma.

## Methods

### Mice

*Anxa5*^−/−^ mice were obtained from Ernst Pöschl and were maintained on a C57BL/6 background; a mixture of genders were used. The mice were genotyped using a mixture of three primers: mA5_Ex3.dw (CGA GAG GCA CTG TGA CTG ACT TCC CTG GAT), mA5_LacZ2.up (GCC AGT TTG AGG GGA CGA CGA CAG), and mA5_Intron3(256).up (CTA GCA GTT GGC CTC ACA CT); *Anxa5*^−/−^ produced a band of ~400 bp and wild type a band of 253 bp. All experiments were performed in accordance with compliance with the Animal Welfare Act regulations and Public Health Service (PHS) Policy; animal research was reviewed and approved by institutional animal care and use committees at Oregon Health & Science University, Michigan State University, and University of Nebraska-Lincoln.

### Immunocytochemistry

Rabbit polyclonal anti-annexin A5 antibody was purchased from Abcam (Cambridge, MA; ab14196). Immunocytochemistry was performed as previously described^36^. Inner ears were harvested and immediately perfused with 4% paraformaldehyde (Electron Microscopy Sciences, Hatfield, PA). The organ of Corti and vestibular end-organs were microdissected in phosphate buffered saline (PBS) pH 7.4 without added MgCl_2_ or CaCl_2_. Saline solutions without added divalents typically have 1–5 μM Ca^2+^, which derives from Ca^2+^ in public water supplies, even after polishing over ion-exchange resins^37^,^38^. Samples were permeabilized with 0.5% Triton X-100 in PBS pH 7.4 and non-specific immunoreactivity was blocked in 5% BSA and 2% goat serum (Invitrogen, Carlsbad, CA) in PBS pH 7.4 (blocking solution) for either one hour at room temperature or overnight at 4 °C. Rabbit polyclonal anti-annexin A5 was diluted 1:200 in blocking solution. Tissues were incubated with primary antiserum for either 2 hours at room temperature or overnight at 4 °C. Polyclonal anti-rabbit IgG secondary antibody conjugated to either Cy3 (Sigma, St. Louis, MO; C2306) or AlexaFluor 488 (Invitrogen, Carlsbad, CA; A11008) was used at either 1:200 (Cy3) or 1:500 (AlexaFluor 488) in blocking solution and incubated for 30 minutes at room temperature. Samples were counterstained with either FITC-phalloidin or rhodamine-phalloidin at 1:200 in blocking solution and DAPI (Invitrogen, Carlsbad, CA) at 1:10,000 in PBS pH 7.4. Samples were imaged using Olympus Fluoview LMS (Center Valley, PA) and either a 60x or 100x oil immersion objective lens. Aside from adjustments to brightness and contrast, no image manipulation was used.

### Shotgun mass spectrometry

Hair bundles were isolated using the twist-off method, as adapted for mouse utricle^14^,^15^,^20^. The utricles were adhered to 35 mm plastic dishes (untreated EASY GRIP Falcon Petri dishes; Becton Dickinson) in Leibovitz’s L-15 Medium without phenol red (21083-027; Thermo Life Technologies). This medium contains 1.26 mM CaCl_2_. After removing otolithic membranes with an eyelash, a plastic washer was placed around the utricles; 4.5% low-melting point agarose in L-15 at 42 °C was added. After the agarose was set at 4 °C for 10–20 min, the utricles were removed from the agarose, leaving bundles in the agarose. To clear away obvious cellular debris, a tungsten needle was used to cut away blocks of agarose; bundles from a single utricle were removed in <0.5 μl agarose plug. Isolated bundles in agarose were frozen at −80 °C, and were pooled later for mass spectrometry analysis. For experiments isolating bundles under low Ca^2+^ conditions, HBSS (Life Technologies 14025-076; 1.26 mM CaCl_2_) and HBSS without calcium and magnesium (HyClone SH30588.02) solutions were substituted for L-15. We used inductively coupled plasma mass spectrometry (ICPMS)^39^,^40^ to measure Ca^2+^ in these solutions. Whole utricles were prepared as described^20^.

Hair-bundle proteins were subjected to in-gel digestion with trypsin as described^3^,^17^,^20^,^41^. Quantitation of proteins of mouse utricle hair bundles at P4–P6 (“P5”) and P21–P25 (“P23”) used MS1 intensity summing with an Orbitrap mass spectrometer^17^,^20^. Technical aspects of these experiments have been described previously^20^; the data are available via ProteomeXchange with identifier PXD002167.

For shotgun analysis of whole utricles, we carried out in-solution tryptic digests of the samples using the enhanced filter-aided sample preparation (eFASP) method^42^. Proteins were digested with 200 ng sequencing-grade modified trypsin (Promega) in the filter unit; a total volume of 100 μl digestion buffer was used and the reaction was carried out at 37 °C for 12–16 hours. After isolating peptides by centrifugation, we extracted them with ethyl acetate to remove remaining deoxycholic acid^42^.

For differential proteomics comparing wild-type and *Anxa5*^−/−^ mice (hair bundles or utricles), protein digests were separated using liquid chromatography with a NanoAcquity UPLC system (Waters), then delivered to an LTQ Velos dual pressure linear ion trap mass spectrometer (Thermo Fisher) using electrospray ionization with a Captive Spray Source (Microm Biosciences) fitted with a 20 μm id taper spray tip. Xcalibur version 2.1 was used to control the system. Samples were applied at 15 μl/min to a Symmetry C18 trap cartridge (Waters) for 10 min, then switched onto a 75 μm × 250 mm NanoAcquity BEH 130 C18 column with 1.7 μm particles (Waters) using mobile phases of water (A) and acetonitrile (B) containing 0.1% formic acid. The gradient was 7–30% acetonitrile over 90 min, and the flow rate was 300 nl/min. A normalized collision energy of 30 was used. Data-dependent collection of MS/MS spectra used the dynamic exclusion feature of the instrument’s control software (repeat count equal to 1, exclusion list size of 500, exclusion duration of 30 sec, and exclusion mass width of −1 to +4) to obtain MS/MS spectra of the ten most abundant parent ions (minimum signal of 5000) following each survey scan from m/z 400–1400. The tune file was configured with no averaging of microscans, a maximum inject time of 200 msec, and automatic gain control targets of 3 × 10^4^ in MS1 mode and 1 × 10^4^ in MS2 mode.

SEQUEST^43^ (version 28, revision 12) was used to search MS2 spectra against version 62 of the Ensembl mouse protein database, with concatenated sequence-reversed entries to estimate error thresholds and 179 common contaminant sequences and their reversed forms. Database processing was performed using custom Python scripts (http://www.ProteomicAnalysisWorkbench.com). SEQUEST searches for all samples were performed with trypsin enzyme specificity. Average parent ion mass tolerance was 2.5 Da. Monoisotopic fragment ion mass tolerance was 1.0 Da. A variable modification of +16.0 Da on methionine residues was also allowed. Peptides identified were SEQUEST and assembled into proteins the PAW pipeline^44^. Proteins were quantified using MS2 intensities, normalized for molecular mass^17^. The ion-trap mass spectrometry proteomics data have been deposited to the ProteomeXchange Consortium^45^ via the PRIDE partner repository with the dataset identifier PXD003005.

To test whether any proteins were differentially expressed in shotgun proteomics experiments, the quantity of each protein was transformed into the logarithm base 2 scale and then normalized by global median normalization^46^. A modified two-sided t-test by empirical Bayes^47^ was used to determine statistical significance between conditions with the false discovery rate^23^ adjustment of p-values for a multiple test correction (wild-type vs. *Anxa5*^−/−^ mice, or Ca^2+^-containing vs. no added Ca^2+^ buffer solutions). Proteins were not filtered by number of identifications per condition, but rather as many proteins as possible were kept as long as the model could be fit. The modified t-test takes advantage of high dimensionality of data and is more suitable for a small sample size than the conventional t-test. The computation was done using the limma package^48^ in R Statistical Computing Environment (www.r-project.org).

### Targeted mass spectrometry

For targeted MS/MS, we carried out in-solution tryptic digests of hair-bundle samples using the enhanced filter-aided sample preparation (eFASP) method^42^. Proteins were digested with 200 ng sequencing-grade modified trypsin (Promega) in the filter unit; a total volume of 100 μl digestion buffer was used and the reaction was carried out at 37 °C for 12–16 hours. After isolating peptides by centrifugation, we extracted them with ethyl acetate to remove remaining deoxycholic acid^42^.

Peptide samples were analyzed with an Orbitrap Fusion Tribrid mass spectrometer (Thermo Scientific) coupled to a Thermo/Dionex Ultimate 3000 Rapid Separation UPLC system and EasySpray nanosource. Samples were loaded onto an Acclaim PepMap C18, 5 μm particle, 100 μm × 2 cm trap using a 5 μl/min flow rate; peptides were separated on a EasySpray PepMap RSLC, C18, 2 μm particle, 75 μm × 25 cm column at a 300 nl/min flow rate. Solvent A was water and solvent B was acetonitrile, each containing 0.1% (v/v) formic acid. After loading at 2% B for 5 min, peptides were separated using a 55-min gradient from 7.5–30% B, 10-min gradient from 30–90% B, 6-min at 90% B, followed by a 19 min re-equilibration at 2% B. Peptides were analyzed using the targeted MS2 mode of the Xcalibur software in which the doubly or triply charged precursor ion corresponding to each peptide was isolated in the quadrupole, fragmented by HCD, and full m/z 350–1600 scans of fragment ions at 30,000 resolution collected in the Orbitrap. Targeted MS2 parameters included an isolation width of 2 *m/z* for each precursor of interest, collision energy of 30%, AGC target of 5 × 10^4^, maximum ion injection time of 100 ms, spray voltage of 2400 V, and ion transfer temperature of 275 °C. No more than 75 precursors were targeted in each run and no scheduling was used. Two to five unique peptides for each protein of interest were chosen for isolation based on previous data-dependent discovery data or from online peptide databases (www.peptideatlas.org, www.thegpm.org). We used the software package Skyline (http://proteome.gs.washington.edu/software/ skyline/) to generate precursor isolation lists for all peptides of interest and export them into the Orbitrap control software.

Skyline was used to analyze targeted MS/MS data. Chromatographic and spectral data from RAW files were loaded into Skyline and manually analyzed to identify fragment ion peaks corresponding to each peptide. RAW files were also processed using Proteome Discoverer (Thermo Scientific) software in order to match MS/MS spectra to an Ensembl spectral database using Sequest HT. Fragment ion peaks for each peptide were chosen according to the following criteria: 1) three or more co-eluting fragment ions contributed to the peak signal, 2) two or more data points were collected across the peak, and 3) one or more spectrum within the peak were matched to correct peptide sequence within the spectral database. If spectra within a specific sample were not identified then a) the retention time of the chosen peak must be within 2 minutes of the retention time of an identified peak for that peptide from another sample and b) the type of daughter ions contributing to the peak must match the identified peptide peak from another sample. If no peak matching these criteria was found in a particular sample the peak area was counted as zero. Chromatographic peak areas from all detected fragment ions for each peptide were integrated and summed to give a final peptide peak area. The peptide peak areas for each protein of interest were averaged for each sample, then averaged for each protein of interest across the biological replicates. The Student’s t-test was used to determine statistical significance between conditions (wild-type vs. *Anxa5*^−/−^ mice, or Ca^2+^-containing vs. no added Ca^2+^ buffer solutions). These targeted data were deposited in the PanoramaWeb^49^ repository at https://panoramaweb.org/labkey/ANXA5.url.

### Protein immunoblotting

To each sample of mouse utricle hair bundles and whole utricles, NuPAGE 4X LDS sample buffer (Life Technologies NP0008), and 0.5 M DTT was added for a final of 1X LDS sample buffer and 50 mM DTT. Samples were heated at 95 °C for 5 min, and resolved using 4–12% SDS-PAGE gels with MES or MOPS buffer (NuPAGE gels and buffers, Life Technologies). Proteins were transferred to PVDF (Immobilon-P, Millipore) and were visualized with India Ink (1:5000) in PBS/0.05% Tween-20. Membranes were blocked with Prime Blocking Agent (GE Healthcare Life Sciences RPN418), and probed with specific primary antibodies which were detected with species-specific HRP-coupled secondary antibodies (Jackson ImmunoResearch AffiniPure 111-035-144, and 115-035-003) and ECL Prime (GE Healthcare Life Sciences RPN2232).

### Vestibular evoked potentials

For VsEP testing, mice were anesthetized with a solution containing ketamine (18 mg/ml) and xylazine (2 mg/ml), using 5–9 μl per gram body weight injected intraperitoneally. Core body temperature was maintained at 37.0 ± 0.1 °C. Linear acceleration pulses, 2 ms duration, were presented to the cranium via a non-invasive spring clip that encircled the head anterior to the pinna and secured the head to a voltage-controlled mechanical shaker. Stimuli were presented along the naso-occipital axis using two stimulus polarities, normal (+Gx axis) and inverted (−Gx axis). Stimuli were presented at a rate of 17 pulses/sec. Stimulus amplitude ranged from +6 dB to −18 dB re: 1.0 g/ms (where 1 g = 9.8 m/s^2^) adjusted in 3 dB steps. Stainless steel wire was placed subcutaneously at the nuchal crest to serve as the noninverting electrode. Needle electrodes were placed posterior to the left pinna and at the hip for inverting and ground electrodes, respectively. Traditional signal averaging was used to resolve responses in electrophysiological recordings. Ongoing electroencephalographic activity was amplified (200,000X), filtered (300 to 3000 Hz) and digitized (100 kHz sampling rate). 256 primary responses were averaged for each VsEP response waveform. All responses were replicated. VsEP intensity series was collected beginning at the maximum stimulus intensity (*i.e*., +6 dB re: 1.0 g/ms) with and without acoustic masking, then descending in 3 dB steps to −18 dB re: 1.0 g/ms. A broad band forward masker (50 to 50,000 Hz, 94 dB SPL) was presented during VsEP measurements to verify absence of cochlear responses.

Latencies and amplitudes of the first peak (P1 and N1) were quantified. P1 for the VsEP is generated by the peripheral vestibular nerve innervating the utricle and saccule. Response peak latencies were defined as the time, in milliseconds, from stimulus onset to the occurrence of each response peak. Peak to peak amplitudes (P1-N1), measured in microvolts, represented the difference in amplitude between the positive peak (P1) and its respective negative peak (N1). Thresholds were defined as the intensity midway between the minimum stimulus intensity that produces a discernable response and the maximum intensity where no response is detectable.

### Auditory brainstem response threshold

The animals were anesthetized with xylazine (10 mg/kg, i.m., IVX; Animal Health Inc., Greeley, CO) and ketamine (40 mg/kg, i.m.; Hospira, Inc., Lake Forest, IL), and placed on a heating pad in a sound-isolated chamber. To ensure the ear canal was free of wax and that there was no canal deformity, inflammation of the tympanic membrane, or effusion in the middle ear, the external ear canal and tympanic membrane were examined using an operating microscope. Needle electrodes were placed subcutaneously near the test ear, both at the vertex and at the shoulder of the test ear side. Each ear was stimulated separately with a closed-tube sound-delivery system sealed into the ear canal. To measure the auditory brainstem response, tone bursts with a 1 ms rise time were applied at 4, 8, 12, 16, 24, and 32 kHz and thresholds obtained for each ear; the tone-burst stimulus intensity was increased in steps of 5 dB. The threshold was defined as an evoked response of 0.2 μV from the electrodes. ABR measurements were taken before noise exposure, 1 hour after noise exposure (temporary threshold shift), and 2 weeks after noise exposure (permanent threshold shift).

### Distortion product otoacoustic emissions

Distortion product otoacoustic emission stimuli consisted of two primary tones, differing 1.2-fold in frequency and both at 60 dB SPL, which were presented over 4–32 kHz. Sound stimuli were generated by 24-bit 192 kHz ESI Waveterminal 192X Sound Card and an in-house acoustic system. DPOAE stimuli were delivered to the ear canal using a coupler tip fitted within the opening of the ear canal to form a closed acoustic system. On the graphs, the amplitude of the 2f1-f2 distortion product was plotted against the f2 frequency where the DP is generated. DPOAE measurements were taken before noise exposure, 1 hour after noise exposure (temporary threshold shift), and 2 weeks after noise exposure (permanent threshold shift).

### Noise exposure

A noise paradigm was used that produces both a temporary change in threshold, to examine reversible loss of sensitivity, as well as a partial permanent threshold change^50^. Mice were put into a small divided wire mesh cage, which was then placed into an open-field acoustic chamber. Mice were exposed to damaging levels of noise for 2 hours. The free field broadband noise level was 0 (control) or 96 dB SPL (acoustic trauma) at 8–16 kHz; a 5 minute ramp-up in noise level was used.

### Data accessibility

Quantitative Orbitrap mass spectrometry data for P5 and P23 mouse hair bundles and utricle have been deposited to the ProteomeXchange Consortium45 via the PRIDE partner repository with the dataset identifier PXD002167. Quantitative ion-trap mass spectrometry data comparing wild-type and Anxa5^−/−^ mice are available via ProteomeXchange with identifier PXD003005. Targeted data were deposited in PanoramaWeb at https://panoramaweb.org/labkey/ANXA5.url.

## Additional Information

**How to cite this article**: Krey, J. F. *et al*. Annexin A5 is the Most Abundant Membrane-Associated Protein in Stereocilia but is Dispensable for Hair-Bundle Development and Function. *Sci. Rep*. **6**, 27221; doi: 10.1038/srep27221 (2016).

## Supplementary Material

Supplementary Information

Supplementary Dataset S1
