# Supplementary material for: Annexin A5 is the Most Abundant Membrane-Associated Protein in Stereocilia but is Dispensable for Hair-Bundle Development and Function
Source: Sci Rep. 2016 Jun 2;6:27221. doi: 10.1038/srep27221 (PMC4890179; doi:10.1038/srep27221)
Supplement: Supplementary Information [file srep27221-s1.doc]

Supplemental Material: Annexin A5 is the Most-Abundant Membrane-Associated Protein in Stereocilia but is Dispensable for Hair-Bundle Development and Function

Jocelyn F. Krey, Meghan Drummond, Sarah Foster, Edward Porsov, Sarath Vijayakumar, Dongseok Choi, Karen Friderici, Sherri M. Jones, Alfred L. Nuttall, and Peter G. Barr-Gillespie

Supplementary Figures and Legends


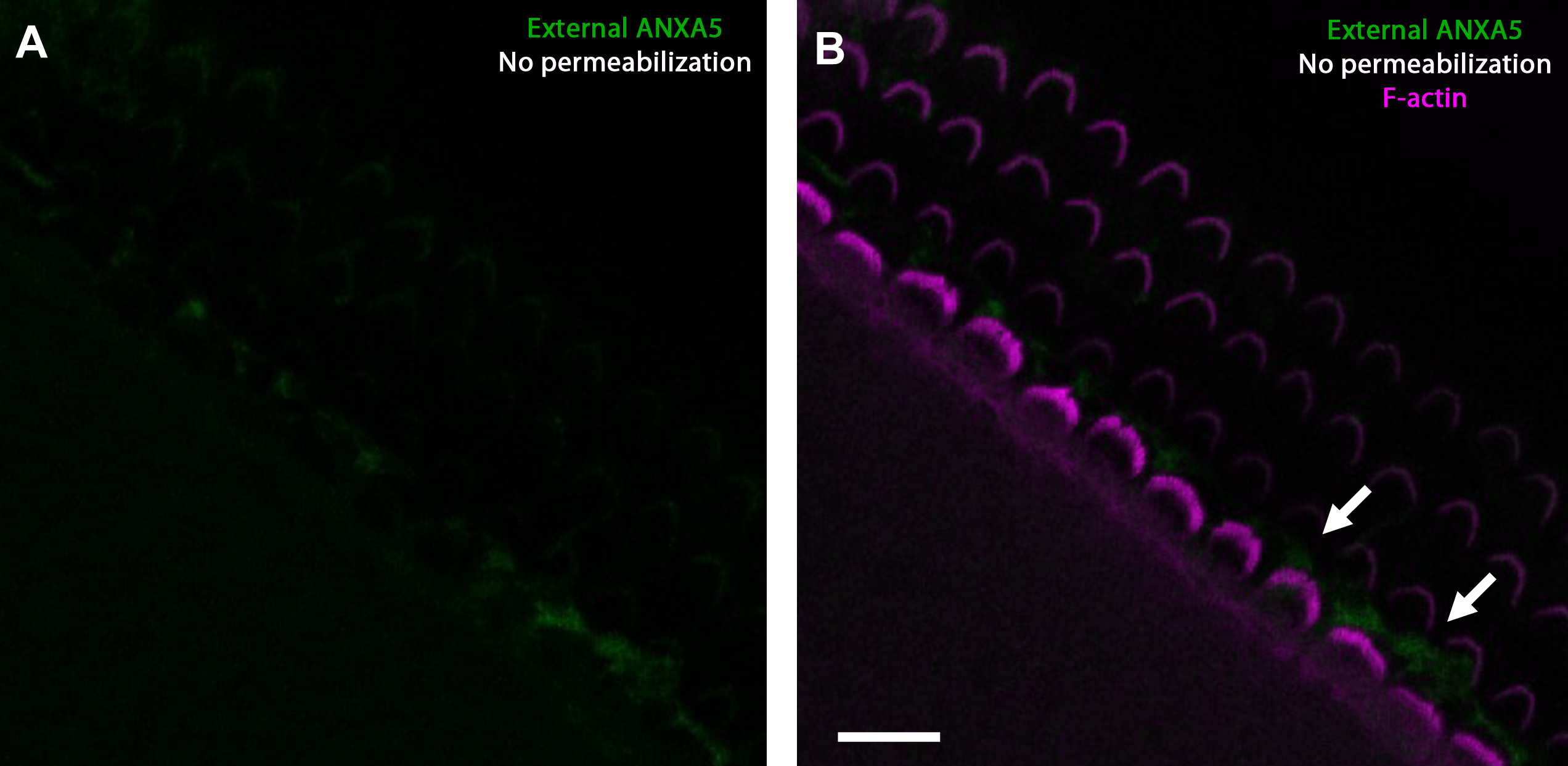


**Supplementary Figure S1.** ANXA5 is not present on the external leaflet of stereocilia membranes. To determine whether ANXA5 in stereocilia is located on the inner or outer leaflet of the plasma membrane, samples were incubated with anti-ANXA5 without permeabilization (A).  To label stereocilia, samples were then fixed a second time to cross link the anti-ANXA5 antibody and then permeabilized and counterstained with phalloidin (B). No ANXA5 immunolabel was seen. Scale bar, 10 µm.


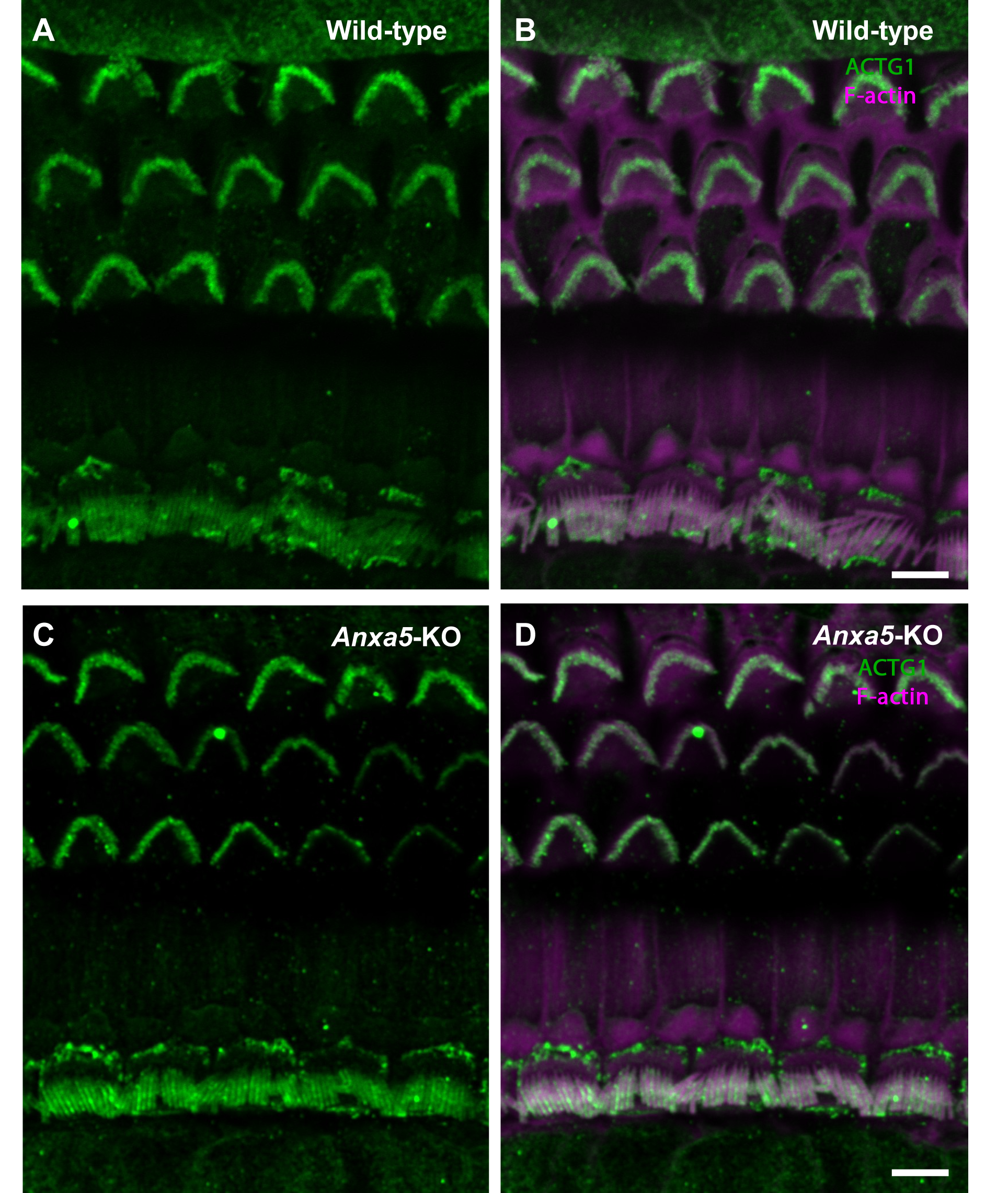


**Supplementary Figure S2.** ACTG1 localizes appropriately in cochlea from *Anxa5-/-* mice. ACTG1 localization in P28 wild-type (**A, B**) and *Anxa5-/-* mice (**C, D**). ACTG1 (green) is enriched around the periphery of the filamentous actin core (red) labeled with rhodamine-phalloidin in merged images (**B, D**). Scale bars, 5 μm.
